# Photobiomodulation Therapy as a Possible New Approach in COVID-19: A Systematic Review

**DOI:** 10.3390/life11060580

**Published:** 2021-06-18

**Authors:** Brenda Thaynne Lima de Matos, Daniela Vieira Buchaim, Karina Torres Pomini, Sandra Maria Barbalho, Elen Landgraf Guiguer, Carlos Henrique Bertoni Reis, Cleuber Rodrigo de Souza Bueno, Marcelo Rodrigues da Cunha, Eliana de Souza Bastos Mazuqueli Pereira, Rogerio Leone Buchaim

**Affiliations:** 1 Department of Biological Sciences, Bauru School of Dentistry (FOB/USP), University of São Paulo, Bauru 17012-901, SP, Brazil; brenda.matos@usp.br (B.T.L.d.M.); karinatorrespomini@gmail.com (K.T.P.); dr.carloshenriquereis@usp.br (C.H.B.R.); cleuberbueno@usp.br (C.R.d.S.B.); 2 Postgraduate Program in Structural and Functional Interactions in Rehabilitation, Postgraduate Department, University of Marilia (UNIMAR), Marília 17525-902, SP, Brazil; danibuchaim@alumni.usp.br (D.V.B.); smbarbalho@gmail.com (S.M.B.); elguiguer@gmail.com (E.L.G.); elianabastosmsn@hotmail.com (E.d.S.B.M.P.); 3 Department of Human Anatomy and Neuroanatomy, University Center of Adamantina (UniFAI), Medical School, Adamantina 17800-000, SP, Brazil; 4 Department of Biochemistry and Nutrition, School of Food Technology of Marília, Marília 17506-000, SP, Brazil; 5 Faculty of Medicine of Jundiaí, Jundiaí 13202-550, SP, Brazil; cunhamr@hotmail.com

**Keywords:** COVID-19, SARS-CoV-2 infection, immunity, inflammation, low-level laser therapy, photobiomodulation therapy, systematic review

## Abstract

COVID-19 is a viral disease characterized as a pandemic by the World Health Organization in March 2020. Since then, researchers from all over the world have been looking for ways to fight this disease. Many cases of complications arise from insufficient immune responses due to low immunity, with intense release of pro-inflammatory cytokines that can damage the structure of organs such as the lung. Thus, the hypothesis arises that photobiomodulation therapy (PBMT) with the use of a low-level laser (LLLT) may be an ally approach to patients with COVID-19 since it is effective for increasing immunity, helping tissue repair, and reducing pro-inflammatory cytokines. This systematic review was performed with the use of PubMed/MEDLINE, Web of Science, Scopus and Google Scholar databases with the following keywords: “low-level laser therapy OR photobiomodulation therapy AND COVID-19”. The inclusion criteria were complete articles published from January 2020 to January 2021 in English. The exclusion criteria were other languages, editorials, reviews, brief communications, letters to the editor, comments, conference abstracts, and articles that did not provide the full text. The bibliographic search found 18 articles in the Pubmed/MEDLINE database, 118 articles on the Web of Science, 23 articles on Scopus, and 853 articles on Google Scholar. Ten articles were included for qualitative synthesis, of which four commentary articles discussed the pathogenesis and the effect of PBMT in COVID-19. Two in vitro and lab experiments showed the effect of PBMT on prevention of thrombosis and positive results in wound healing during viral infection, using the intravascular irradiation (ILIB) associated with Phthalomethyl D. Two case reports showed PBMT improved the respiratory indexes, radiological findings, and inflammatory markers in severe COVID-19 patients. One case series reported the clinical improvement after PBMT on 14 acute COVID-19 patients, rehabilitation on 24 patients, and as a preventive treatment on 70 people. One clinical trial of 30 patients with severe COVID-19 who require invasive mechanical ventilation, showed PBMT-static magnetic field was not statistically different from placebo for the length of stay in the Intensive Care Unit, but improved diaphragm muscle function and ventilation and decreased the inflammatory markers. This review suggests that PBMT may have a positive role in treatment of COVID-19. Still, the necessity for more clinical trials remains in this field and there is not sufficient research evidence regarding the effects of PBMT and COVID-19 disease, and there is a large gap.

## 1. Introduction

COVID-19 (SARS-CoV-2) is a viral disease discovered in December 2019, in the Chinese city of Wuhan, and spread to several continents, which led the World Health Organization (WHO) to characterize the disease as a pandemic 11 March 2020 [1]. This fact demonstrates the considerable speed of propagating this infectious pathogen, spreading around the world in a few months because on average, one infected person spreads the infection to three other people [2]. The forms of contamination are varied, such as through droplets of saliva, contaminated objects and surfaces, coughing, and sneezing. Thus, social distance and hygiene measures are essential to reduce contamination by the virus, with social isolation being the most effective method [3,4].

The most common symptoms of COVID-19 are cough, fever, anosmia, ageusia, difficulty breathing, and in more severe cases, pneumonia. Some conditions, such as diabetics, cardiac and hypertensive individuals, are more susceptible to presenting more severe conditions. This is also observed in people with low immunity since immunity is a great ally to the fight against coronavirus in the human body [5,6].

However, although high immunity is a good ally, its low levels can intensify the inflammation process. In the case of a defective immune response against COVID-19, an accumulation of immune cells in the lungs can occur, causing an exacerbated production of pro-inflammatory cytokines, which can damage the lung structure. With this excess of cytokines, it can spread to other locations, causing damage to multiple organs. In a healthy immune response, virus-specific T cells target inflammation and eliminate infected cells, preventing the virus from spreading, leading to minimal damage. Thus, stimulation of immunity is critical to approach the new coronavirus [7].

There are several ways to increase immunity that can be conducted by practicing physical activity and having good eating habits, which have been shown to affect immune sensitivity and inflammation [8]. There is also the possibility of using Vitamin D and Zinc in capsules, which in the body have beneficial immunomodulatory and anti-inflammatory effects in infections caused by viruses. Still, in the context of COVID-19, there is no scientific evidence to prove the effectiveness of these elements [9].

Another way of modulating the immune system is the use of photobiomodulation therapy (PBMT), which can stimulate immune cells to clump together at the site of the affected cells and assist them in survival through greater anti-inflammatory cytokine expression and decreased pro-inflammatory. In precursor studies, it was observed that laser light in low doses accelerated the healing of excisional wounds, called laser biostimulation [10]. There was a clear lack of consensus on the terminology for this therapy. The term most often used is low-level laser therapy (LLLT). ‘‘Laser’’ is not the most appropriate term as other light devices such as LEDs are currently used for this application. The ideal term and its definition must be precise and emphasize its scientific basis, with photobiomodulation being the term of choice to describe this use, meaning light therapy that uses non-ionizing forms of sources including lasers, LEDs, and broadband light in the spectrums visible and infrared [11].

The effect of PBMT is due to its absorption by tissues through photoreceptors, facilitating events such as mitochondrial respiration, calcium transport—which results in more significant cell proliferation—repairing, and regenerating tissues [12,13,14,15]. PBMT has to assist in the recovery process from nerve [16,17,18], bone [19,20,21], respiratory tract [22,23,24,25], and other injuries involved in functional rehabilitation, favoring the patient’s recovery. LLLT can be used with the ILIB technique (Intravascular Laser Irradiation of Blood), which increases immunity, inducing positive effects on the expression of immunoglobulins (IgA, IgM and IgG) and modulation of inflammation. Therefore, this technique can be used to treat various pathogens such as infectious diseases, bronchitis, and pneumonia [26]. 

Given the great importance of biological mechanisms for the prevention, treatment, and rehabilitation of diseases such as COVID-19, the aim of this systematic review was to analyze the use of PBMT as a possible new approach as a complementary therapy in the treatment of patients affected by COVID-19.

## 2. Materials and Methods

Four databases were searched, PubMed/MEDLINE, Web of Science, Scopus, and Google Scholar, using the following as keywords: “low-level laser therapy OR photobiomodulation therapy AND COVID-19”. The collection of articles was carried out considering the period of publication from January 2020 to 31 January 2021, by two evaluators previously used to the evaluation of the criteria to avoid bias in the selection.

### 2.1. Inclusion Criteria

- Full-text articles;
- English language.

### 2.2. Exclusion Criteria

- Editorials;
- Review articles;
- Brief communications;
- Letters to the editor;
- Comments;
- Congress abstracts;
- Articles that do not provide full text;
- Other languages than English.

The studies that presented titles and abstracts related to the theme of the initial research were verified, using the variables low-level laser therapy or photobiomodulation therapy and their use in the pandemic resulting from the new coronavirus SARS-CoV-2 (COVID-19). The next step was to evaluate the full text of the articles previously selected by the abstract. The methodology, results, and relevance were considered for listing the choice of articles.

For inclusion in the research, the articles were necessarily accessed in their full content. Analysis and synthesis of reflective and consistent texts on the subject were carried out.

**Figure 1:**
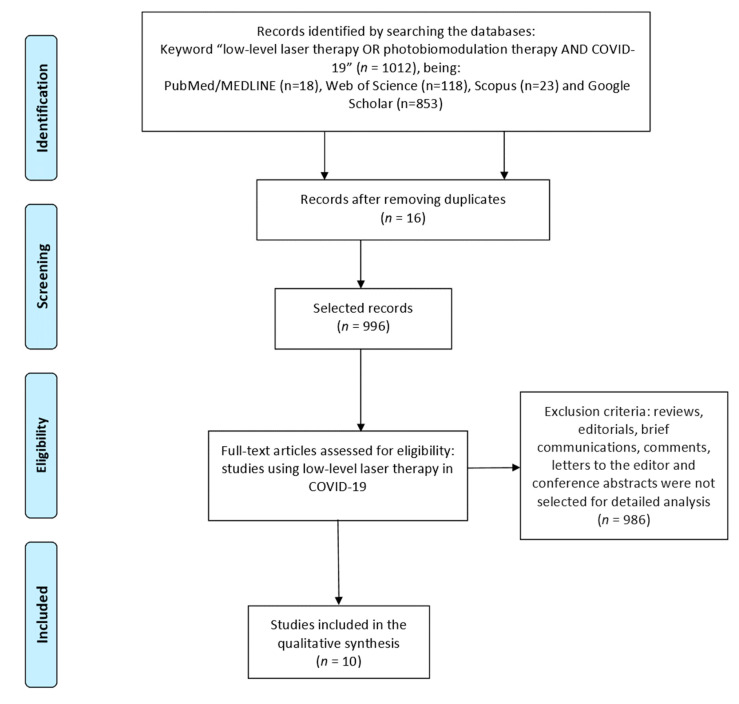
Flow diagram showing the study selection [27].

The phases of the bibliographic search were organized by the Prisma Flow Diagram [27] (Figure 1).

## 3. Results

The bibliographic search found 18 articles in the Pubmed/MEDLINE database, of which 13 were excluded because they were outside the eligibility criteria. We also found 118 articles on the Web of Science and selected one article (after excluding duplicates), 23 articles on Scopus and selected one article, and, finally, 853 articles on Google Scholar and selected three articles.

The articles selected to compose this systematic review are shown in Table 1.

## 4. Discussion

This systematic review was carried out to evaluate the published literature, against the established descriptors and the inclusion and exclusion criteria of the articles, in an attempt to identify patterns and trends on the association of COVID-19 and PBMT, as well as to identify possible gaps and trends new areas of research. Although there are still few studies, our results shown that PBMT can bring benefits for COVID-19 patients since it is related to modulating the immune system, to the reduction of inflammation, and other beneficial activities for the restoration of health.

SARS-CoV-2 leads to the death of cells attacked by it, causing a blockage in the airways with its waste, making breathing more difficult with the accumulation of dead cells and fluids in the lungs [38]. The immune reaction in the infected patient usually causes inflammation and fever. With vasodilation at the site of infection, the lungs accumulate fluid [39]. This exaggerated immune response is called cytokine storm, which causes Acute Respiratory Distress Syndrome (ARDS), fever, multiple organ failure, and death [33]. The effect of laser therapy is related to the amount of laser energy per cm^2^. The minimum therapeutic dose for a biostimulant effect for the red and infrared laser is 0.01 J/cm^2^, while for the blue, ultraviolet, and green lasers it is 0.001 J/cm^2^. The effective stimulation dose is 1 J/cm^2^ and doses greater than 10 J/cm^2^ can produce inhibitory effects, used in conditions that require inhibition and suppression, such as, for example, suppressing the inflammatory response in an attempt to avoid or minimize the storm of pro-inflammatory cytokines [33].

The use of phototherapy in treating illnesses has been reported since Ancient Greece, initially called Heliotherapy, and consisted of leaving the sick exposed to the sun to cure their ailments, as shown in Table 1 [29]. In 1918, during the crisis caused by the Spanish flu, phototherapy proved to be a great ally in the treatment of this disease, being pointed out as one of the most significant factors in reducing mortality. Since then, several studies have been carried out to understand better the beneficial effects of light, especially blue, violet, red, and infrared light [29]. 

These studies found blue and violet light effects such as virus inactivation, including coronavirus and the common flu virus, and antimicrobial effect. These effects can be used, respectively, for cleaning, avoiding contagion by a coronavirus from infected surfaces, and mitigating opportunistic bacteria in the treatment of COVID-19 [29,40,41]. In addition, therapeutic effects of red and infrared light have also been found effective in treating various diseases such as pneumonia, decreasing inflammation, pulmonary edema, and fibrosis in the lung. These effects are important to avoid many complications of COVID-19, with ARDS the leading cause of death from the disease [29]. From this, more technological and specific lasers were developed, giving rise to photobiomodulation, which can be a great ally to the treatment of COVID-19.

**Figure 2:**
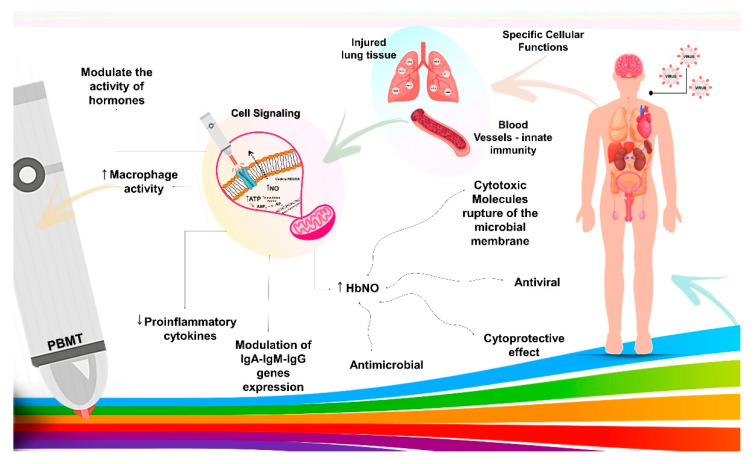
The schematic diagram illustrates COVID-19 infection in the human body, followed by rapid spread from viral sanctuary sites. Early innate host immune response dictates viral load at the acute phase. Photobiomodulation therapy (PBMT) acts on transmembrane receptors present in the mitochondria with specific cellular functions, modulating cells with functional deficits, such as blood cells and lung tissue, promoting signaling by the absorptivities of electromagnetic rays. These chromophores convert electromagnetic energy into adenosine triphosphate, and then induce increased macrophage activity, modulation of plasma hormone levels, decreased proinflammatory cytokines, modulated expression of IgA, IgM, and IgG immunoglobulins, and increase of the HbNO synthesis. The results are positive with the synthesis of cytotoxic molecules to microbial membranes, which leads to the destruction of microorganisms of all types in the blood and cytoprotective effect to human cells.

Moreover, the effects of the blue laser go beyond the antimicrobial and anti-inflammatory capabilities. Its irradiation in the blood also favors hormonal harmonization, pain reduction, and the release of hemoglobin nitric oxide (HbNO), which has a powerful antibacterial effect and causes the destruction of microorganisms of all types in the blood. This can be used in antimicrobial photodynamic therapy with the combination of Riboflavin, a photosensitizer, for bacterial, viral, and parasitic diseases. The production of nitric oxide (NO) in the immune system is key to the capabilities and characteristics of immune cells and having cytotoxic and cytoprotective, antiviral, antimicrobial effects, and stimulating and suppressing the immune system. With the blue laser increasing the level of NO, mitochondrial biogenesis in different types of cells and oxygen connection to red blood cells is also increased (Figure 2) [5,30,39]. However, the use of lasers in viral mortality is not widespread, but there is evidence that the 405 nm LED light has antiviral activity [29]. Thus, the coronavirus could be killed or altered to be used in the preparation of vaccines. For this, the maximum irradiation takes 10 min with a power of 2 mW [5,30].

Since the onset of the disease, there is a consensus among clinicians and researchers about the role of the SARS-CoV-2 virus in pulmonary complications, which can lead to a severe respiratory syndrome. Nitric oxide (NO) is beneficial in low concentrations, but it can produce reactive nitrogen species (RNS), such as peroxynitrite, in high concentrations. Both ROS (species that react to oxygen) and RNS are destructive at high doses. In pulmonary inflammation, the increased influx of neutrophils produces high levels of ROS and RNS, damaging the lung tissue (Figure 2). PBMT can reduce ROS formation [42,43], reduce pulmonary edema, neutrophil influx, and promote lung tissue regeneration and better oxygenation to all related organs [44].

As for the type of laser, infrared is preferred due to the greater capacity of penetrating the lung tissue [45]. The dosimetry of adequate energy density is 9.5–10.5 J/cm^2^. Continuous irradiation at different points in the respiratory system can be helpful in the treatment of COVID-19 pneumonia. PBMT can be used as a preventive approach in high-risk, elderly, or comorbid patients receiving pre-treatment PBMT while still at an early stage of the disease [40]. In addition, PBMT can be considered a therapeutic approach in hospitalized patients before their condition worsens enough to require ICU admission [44].

Moreover, in addition to the damage to lung tissues, SARS-CoV-2 also favors the formation of thrombi. Nitric oxide has the potential to treat thrombosis associated with mechanical devices due to its ability to reduce platelet activation and due to the central role of platelet adhesion in device thrombosis [28]. One study, shown in Table 1, reports the effects of nitrite, distant red light, and their combination in various measures of blood clotting using a variety of agonists. The authors concluded that the combination of extreme red light and nitrite treatment decreased the coagulation measures in all cases [28]. It is believed that the radiation from the infrared laser near the red (R/NIR) can stabilize the membrane of red blood cells (RBC) by increasing its resistance to destructive factors, reducing trauma in the use of equipment, such as extracorporeal circulation [41].

Likewise, there are techniques for increasing the efficiency of light, such as PDT, since its penetration into tissues is limited. One technique is the association of PDT with phototherapy agents, which sensitize specific molecules to absorb light better. PDT has been clinically validated in several esophageal pathologies and some lung cancers and can be used to destroy pathogenic microorganisms such as bacteria and viruses [29,46]. This can also be seen in the association of PDT with Phthalocyanines, a phototherapeutic agent that makes incorporation by target cells easier. The use of phototherapeutic agent Phthalomethyl D shows promising effects if used with the aim of leading to immunological improvement, cure, and repair against viral induction [32].

In this scenario, with the use of PDT by the ILIB technique through radial artery for 30 min at a wavelength of 660–460 nm, 100 mW output power, and in continuous wave mode for point irradiation (Ecco Fibras, São Paulo, SP, Brazil) and the use of Phthalomethyl D administered orally or introduced into the digestive tract, wound healing and the systemic inflammatory process were improved. It can be suggested that, at low doses, phagocytic activity increases, and cell viability decreases after the execution of this technique. Thus, the relationship between the action of the laser and the activation of monocytic cells may explain the process of resolving systemic inflammation with more efficient wound closure. Thus, its contribution to the anti-inflammatory response during viral infection may be an approach to SARS-CoV-2 [32].

Clinical study using PDT in 300 patients, methylene blue (MB) photosensitizer and 660 nm red light applied in the oral and nasal cavity, compared to placebos, led to significant decreases in morbidity and reduced mortality rates, contributing to the immune response [47]. In vitro, PDT with MB and Radachlorin, using continuous laser with wavelength 662 nm at doses of 16 J/cm^2^ and 40 J/cm^2^ of laser irradiation, shows high antiviral activity against SARS-CoV-2 [48].

Furthermore, COVID-19 mainly attacks the lung and heart tissues. This is because the coronavirus binds to the cell through the angiotensin-converting receptor, which is highly expressed in lung and heart cells. Thus, it is vital that the receptor is not available to bind to the coronavirus, and for that, Vitamin D, which also binds to that receptor, can help. There are no acute cases of heart attack at balanced levels of Vitamin D due to COVID-19 [31]. In addition, Vitamin D has a broad spectrum of immunomodulatory, antifibrotic, anti-inflammatory and antioxidant actions [9,49].

One of the safest ways to increase Vitamin D levels in the body is the use of the 50 mW 589 nm yellow laser, which can be applied through the nose or acupuncture points (acupuncture laser) [31]. In addition, Vitamin D deficiency has been found, to be related to increased mortality and disease progression. It was also observed that the expression of inflammatory cytokines was inhibited by Vitamin D, and its insufficiency was related to the overexpression of cytokines and that it plays an important role in cardiovascular diseases and diabetes mellitus, risk factors for the disease [45]. Therefore, ways to obtain good Vitamin D levels in the body can be a preventive approach to complications of COVID-19 [31,49].

In severe SARS-CoV-2 infections, recent histopathological studies have emphasized the important role of Endothelial Cells (EC) in immune-thrombosis, vascular dysfunction, and inflammation. The storm of pro-inflammatory cytokines, commonly seen in irregular immune responses, can also cause endothelial dysfunction (EnD). The endothelitis caused by COVID-19 can explain the impairment of the systemic microcirculatory function of different organs and is highly harmful [50]. The endothelium performs multiple functions, such as regulating the transport of biologically active substances, barrier, participation in phagocytosis, and control of the diffusion of fluids, electrolytes, metabolic products, and platelet adhesion. Therefore, EnD can be catastrophic, becoming a primary cause of high mortality and the development of diseases or complications that disrupt a fully human life, so preventing the development of EnD is paramount [37,50].

Thus, with LLLT, at the systemic level, it is possible to activate microcirculation and metabolism, better regeneration of lung tissue, increased local immunity, and improved muscle support for the respiratory act. As a result, laser illumination using the ILIB technique has been widely used to correct endothelial function. The “classic” way of using this method is with a wavelength of 635 nm, 2–3 mW of lighting power at the fiber outlet, 10–20 min of exposure. With this technique, in seven daily sessions, patients with mild illness (six people) achieved an overall improvement in health, relief of chest pain during cough, and improvement in sputum discharge due to the increased effectiveness of the cough impulse [37,51].

One patient, with a severe disease course, used five combined laser therapy procedures: ILBI-525 intravenous laser blood illumination + LUVBITM violet laser ultra-blood illumination (525 nm wavelength, green spectrum, 2 mW lighting power, 5 min exposure per zone + 365 nm wavelength, UV spectrum, 2 mW of illumination and 5 min exposure per zone on alternate days) and exposure to pulsed IR LLLT (904 nm wavelength with light pulse duration of 100 ns, pulsed power of 15 W, power density of 10–15 W/cm^2^, frequency of 80 Hz and 1.5 min of exposure). With this approach, in the fifth procedure, the patient noticed a significant improvement in general health and the disappearance of shortness of breath with moderate physical effort [37,51,52].

Knowing that LLLT is effective against the cytokine storm and ARDS, promoting tissue healing and regeneration, experimental models in animals have shown a decrease in inflammation without impairing lung function in the case of acute lung injuries, which could be an approach to pulmonary inflammatory diseases [25]. In this experimental study, the effect of LLLT on chronic obstructive pulmonary disease was evaluated. The results demonstrated that LLLT significantly reduced the number of inflammatory cells and the secretion of pro-inflammatory cytokines as IL-1 β, IL-6, and TNF-α in bronchoalveolar lavage (BAL). It was also observed that LLLT decreased collagen deposition and the expression of the purinergic P2X7 receptor. Thus, LLLT is considered a promising treatment for other lung diseases, such as COVID-19 [25].

In addition, prolonged time on ventilators causes lung injuries, which can further aggravate the disease. For this, the use of LLLT is shown to minimize this side effect. This was proven in experimental models of ventilator-induced lung injury in rats, and with the use of LLLT there was an anti-inflammatory effect via decreased lung injury scores and lower neutrophil counts in alveoli, interstitial, and bronchial lavage [42]. Modulation of inflammatory factors and a drive for healing are needed to help patients get off the ventilators. Thus, LLLT is a safe and non-invasive technique that has been used for decades in the treatment of pain, wound healing, and health conditions, including diseases of the respiratory system. LLLT combined with standard medical care to optimize response to treatments, reduce inflammation, promote healing, and speed recovery times is a promising approach [33].

Another technique that can be useful in this scenario is PBMT when used alone or combined with the static magnetic field (PBMT-sMF). In a study, with patients with severe COVID-19 that required mechanical ventilation were randomly assigned to receive PBMT-sMF (six sites on the lower chest—189 J in total and two sites in the neck area—63 J in total) or PBMT-sMF placebo daily for the entire ICU stay, it was noted that PBMT-sMF was able to improve some ventilatory parameters, in addition to the infectious process, and immune response [36]. Thus, these techniques suggest that treatment with PBMT-sMF or LLLT may reduce the burden on the hospital and health systems and the use of scarce health care resources during this pandemic [33,36].

PBMT techniques in the hospital were observed in the clinical case of patient with severe COVID-19 [34]. This was the case of a 32-year-old Asian woman with morbid obesity and a body mass index of 52, and a history of excision of meningioma and asthma presented to the emergency room with a positive COVID-19 test and shortness of breath, cough, and diarrhea. The patient presented hypoxia with oxygen saturation via pulse oximetry (SpO2) of 88% in room air, tachypneic with a respiratory rate of 35, feverish temperature of 100.5 ºF, pulse rate 89, and blood pressure 106/84. The condition was considered severe, and was worsened, leading to her admission to the ICU. As a result, treatment with LLLT was started. During the first laser treatment, her SpO2 increased from 92% to 97% at 3 L/min of oxygen within 10 min of starting treatment. After the second laser treatment, the patient was breathing without dyspnea. After treatments, her respiratory rate returned abnormally 19–20 breaths/min. After the fourth treatment, the patient was able to ambulate independently and improved the ability to perform activities of daily living. The patient was discharged two days after her last treatment with 1 L/min of oxygen [34].

Another case was that of a 57-year-old African American man with severe COVID-19 who received four sessions of PBMT once a day by a laser scan and with pulsed modes of 808 nm and super pulses of 905 nm for 28 min [35]. Oxygen saturation (SpO2) increased from 93–94% to 97–100%, while oxygen demand decreased from 2–4 L/min to 1 L/min. The RALE score improved from 8 to 5. The Pneumonia Severity Index improved from Class V (142) to Class II (67). Additional pulmonary indium (Brescia-COVID and SMART-COP) decreased from 4 to 0. CRP normalized from 15.1 to 1.23. This patient tolerated all 4 treatments daily and observed a significant improvement in breathing immediately after each treatment. Paroxysmal coughing fits were resolved after the third treatment. Upon completion of the fourth treatment, the patient was able to ambulate with physical therapy. The next day after his final treatment, the patient was discharged to an acute rehabilitation facility with oxygen at 1 L/min [35]. 

Perhaps soon, the results of clinical trials currently underway should contribute to the assessment of PBMT efficacy for improving respiratory, inflammatory, coagulation and morbidity-mortality parameters in patients undergoing these studies.

## 5. Conclusions

The use of photobiomodulation therapy, through the low-level laser, in the initial stage of COVID-19, which can evolve to more severe stages, has been shown to be beneficial in treating the disease complications. Clinical trials and studies with a larger sample population are needed to confirm this approach, both in terms of prevention and treatment assistance. Photobiomodulation is promising as an approach to COVID-19 because previous studies show its effectiveness, mainly in reducing inflammation levels, which may favor the control of the exacerbated reaction caused by SARS-CoV-2, mainly in the lungs.

## Figures and Tables

**Table 1:** Articles selected according to the eligibility criteria.

Reference	Type of Article	Human/Animal	Intervention	Results	Conclusions
Wajih et al., 2021 [28]	Experimental, in vitro research	Human blood	Evaluation of nitrite, extreme red light, and its combination on various measures of blood clotting using a variety of agonists. Platelet-rich plasma turbidity assays, platelet activation using flow cytometry analysis of a fluorescently labeled antibody to the activated platelet fibrinogen binding site, platelet aggregometry based on multiple impedance, and evaluation of platelet adhesion to collagen coated flow-through microslides.	The combination of extreme red light and nitrite treatment decreased the coagulation measures, but in some cases, the mono-treatment with nitrite or light alone showed no effects. It was observed that platelet adhesions were inhibited by the combination of nitrite and light treatment, while nitrite alone and extreme red light alone led to decreased adhesion.	These results support the potential of the combined treatment of extreme red light and nitrite for the prevention of thrombosis in extracorporeal devices or with shallow-tissue depth, where extreme red light can penetrate. Given the role of thrombosis in COVID-19, application to the treatment of patients infected with SARS-Cov-2 can also be considered.
Enwemeka et al., 2020 [29]	Perspective study	Not applicable	This study relates phototherapy throughout history, initially called heliotherapy, where sunlight was used as a treatment for various diseases, which was of great help in the 1918 pandemic against the Spanish flu. It also reports the beneficial effect of blue/violet light, red and infrared light. With the development of lasers and the evolution of phototherapy, laser therapy originated and is currently called photobiomodulation.	It was observed inactivation of many viruses, including the common flu coronavirus, and antimicrobial effect. Red and infrared light have important therapeutic value, as they are effective in the treatment of various diseases, reducing, for example, inflammation and fibrosis in the lung.	Blue/violet light has potential use in treatments, mitigating opportunistic bacteria, and aiding in hygiene. The effects of red light are effective in treating complications of COVID-19, such as inflammation, pulmonary edema, pneumonia, and acute respiratory distress syndrome (ARDS), which are its leading causes of death.
Razzaghi and Kamani, 2020 [30]	In vitro study	Human	The authors defend the use of the blue laser, which has antimicrobial and anti-inflammatory effects, positive for the immune system. Blood irradiation has a positive influence on the release of hemoglobin nitric oxide (HbNO), with antibacterial effects. This can be used for Photodynamic Therapy (PDT) in combination with Riboflavin as a photosensitizer in order to reduce inflammation of the lungs, increase the amount of nitric oxygen, which will increase the body’s immunity, and improve the supply of oxygen to the blood and tissues, used as a treatment for COVID-19.	It has been observed that LED light has an antiviral effect and that nitric oxide production is the key to many of the capabilities and characteristics of immune cells, such as dendritic cells, Natural Killer cells (NKs exterminators), mast cells, macrophages and other phagocytes and has a cytotoxic and cytoprotective, antiviral effect, antimicrobial, stimulating and suppressing the immune system.	With the results on blue light and the effects of nitric oxide on the immune system along with the emergence of a treatment for COVID-19, the authors discussed a preventive approach using these devices. The form and possible beneficial effects for the treatment of COVID-19 have been described for a laboratory study to be carried out.
Kamani, 2020 [31]	In vivo study	Human	In this study, this was associated with the LLLT and acupuncture technique. The yellow laser light increases Vitamin D and serotonin levels through application to the nose and acupuncture points. The laser acupuncture method has been irradiated perpendicularly to the top of the skin and is a safer way than taking Vitamin D tablets.	The results were different at different acupuncture points. For example, at LR14, activation of the temporal cortexes, frontal cortex, right parietal cortex, and deactivation in the temporal cortex in the bilateral superior temporal gyrus and the limbic cortex. In KI3, there was no significant activation or deactivation.	The author took a preventive approach recognizing the properties of light and studies in this area and its possibility of becoming a treatment for COVID-19.
Pinto et al., 2020 [32]	Experimental, in vivo research	Animal/mice	This study aimed to characterize the PDT associated with the phototherapeutic agent Phthalomethyl D in the process of immunological improvement, cure and repair against SARS-CoV-2. The study used 69 rats submitted to surgery for viral induction and separated into two groups, one treated with ILIB laser and the other with this technique added to the association of Phthalomethyl D.	As a result, both groups achieved a high rate of partial incision closure and acute inflammatory control. In the group treated with the ILIB technique associated with Phthalomethyl D, there was a greater amount of fundamental amorphous substance, fibrocytes, fibroblasts, and giant cells, and the number of keratinocytes, the amount of keratin and the epidermal thickness was reduced concerning the group treated only with the ILIB technique.	Thus, with positive results using the ILIB technique associated with Phthalomethyl D, which obtained satisfactory results in increasing healing, repair, and immunomodulation during viral infection, contributing to the anti-inflammatory response, the use of this treatment in the approach to SARS-CoV-2 is possible.
Mokmeli and Vetrici, 2020 [33]	Study based on clinical experience, peer-reviewed studies and solid laboratory data in experimental animal models.	Not applicable	This study aimed to provide a protocol for the use of low-level laser therapy (LLLT) to prevent disease progression, minimize the time required to use a ventilator, improve healing and shorten recovery time. This technique was chosen because it has strong anti-inflammatory effects and attenuates the cytokine storm and being a safe, effective, low-cost technique with no side effects.	LLLT has therapeutic potential for the respiratory syndrome, with the ability to reduce pain and heal wounds in health areas. In addition, it is also used to treat pneumonia by veterinarians. Based on clinical experience, peer-reviewed studies, and laboratory data in experimental animal models, LLLT mitigates the cytokine storm at various levels and reduces inflammatory metabolites.	As the mortality of COVID-19 is closely related to respiratory syndrome and cytokine storm, in addition to the presence of lesions in the lungs caused by the use of a ventilator for longer than expected, LLLT is a resource that can be associated with the treatment and recovery COVID-19, still needing clinical trials to assess this approach objectively.
Sigman; Mokmeli; Vetrici, 2020 [34]	Case report	Human	This case report demonstrated the use of low-level laser therapy (LLLT) applied to a 32-year-old patient, morbidly obese with severe COVID-19. The patient received four sessions once a day using a laser scanner. Pulsed laser beams of 808 nm and 905 nm were applied to the posterior part of the chest for 28 min. Before treatment, oxygen saturation (SpO2) via pulse oximetry was 88–93% with 5–6 L of oxygen.	The patient was evaluated before and after LLLT and showed significant improvement. Increasing the SPO2 increased to 97–99% with 1–3 L of oxygen. The RALE score was reduced from 8 to 3, Brescia COVID from 4 to 0, and SMART-COP from 5 to 0. Interleukin-6 decreased from 45.89 to 11.7 pg/mL, ferritin from 359 to 175 ng/mL and CRP from 3.04 to 1.43 mg/dL. In addition, the patient noticed a considerable improvement in respiratory symptoms.	Due to the improvement in the respiratory indexes in a few days, radiological findings and inflammatory markers, the case report suggests that the adjunctive LLLT may be an ally to the conventional treatment of patients with severe COVID-19 and morbid obesity.
Sigman et al., 2020 [35]	Case report	Human	In this case report of a severe case of pneumonia caused by COVID-19, PBMT was used in in a 57-year-old man who received four sessions of PBMT once a day using a pulsed 808 nm and super laser scanner pulsed at 905 nm for 28 min. The patient was evaluated before and after treatment utilizing radiological assessment of lung edema (RALE), pulmonary severity indices, blood tests, oxygen requirements, and questionnaires.	After treatment, oxygen saturation increased from 93–94% to 97–100%, while the oxygen requirement decreased from 2–4 L/min to 1 L/min. The RALE score improved from 8 to 5. The Pneumonia Severity Index improved from Class V (142) to Class II (67). The additional pulmonary indices (Brescia-COVID and SMART-COP) decreased from 4 to 0. CRP normalized from 15.1 to 1.23. In addition, the patient reported significant improvement in the Community-Acquired Pneumonia assessment.	With PBMT, respiratory rates, oxygen requirement, and radiological findings improved significantly over the days, and there was no need for a ventilator, suggesting the use of PBMT as a complementary treatment for patients with pneumonia due to COVID-19, requiring further clinical trials to assess the effects.
Marchi et al., 2020 [36]	Randomized placebo-controlled trial research	Human	This study investigated whether PBMT-sMF is able to decrease the length of stay in the Intensive Care Unit (ICU) and to reduce the mortality rate of patients with severe COVID-19 who require invasive mechanical ventilation. Patients were randomly assigned to receive PBMT-sMF (6 sites on the lower chest—189 J in total and 2 sites in the neck area—63 J in total) or PBMT-sMF placebo daily for the entire ICU stay.	The 30 patients were submitted to randomization (15 receiving PBMT-sMF and 15 placebos). There was no statistical difference between the groups for the length of stay in the ICU. Regarding secondary studies, statistical differences were observed in favor of PBMT-sMF for diaphragm thickness, the fraction of inspired oxygen, partial pressure of oxygen/fraction of inspired oxygen, reactive protein C, lymphocyte count, and hemoglobin.	The study demonstrated that for patients with severe COVID-19 who require invasive mechanical ventilation, PBMT-sMF was not statistically different from placebo for the length of stay in the ICU, but improved diaphragm muscle function, ventilation and led to decreased levels of C-reactive protein (PCR) and hemoglobin count, in addition to increased lymphocyte count.
Moskvin; Askhadulin; Kochetkov, 2021 [37]	Review and experimental, in vivo research	Human	106 patients were treated at two health centers in Russia. A total of 22 had SARS pneumonia and were admitted for pulsed infrared rehabilitation; 14 patients had acute forms of COVID-19 and were treated with the LASMIK device, wavelength 904 nm, pulsed mode, externally and ILBI-525 (intravenous laser blood illumination) + LUVBI (ultraviolet laser blood illumination); 70 people have taken preventive non-invasive LLLT courses.	LLLT is effective in preventing the development of endothelial dysfunction. Clinically, there was good tolerability of the treatment and improvement in sputum discharge and general health. In the fifth procedure, the severity of general hypoxia decreased, and in the prevention procedures, there was good tolerance, and there were no cases of COVID-19.	LLLT is a treatment method that promotes lung tissue regeneration and mitigates the consequences of COVID-19. Thus, LLLT can be used for both prevention and treatment in patients with COVID-19.

## Data Availability

Not applicable.
